# Bodybuilding: A Comprehensive Review of Performance-Enhancing Substance Use and Public Health Implications

**DOI:** 10.7759/cureus.41600

**Published:** 2023-07-09

**Authors:** Saket Mantri, Sristy Agarwal, Arpita Jaiswal, Seema Yelne, Roshan Prasad, Mayur B Wanjari

**Affiliations:** 1 Medicine, Jawaharlal Nehru Medical College, Datta Meghe Institute of Higher Education and Research, Wardha, IND; 2 Medicine, Jawaharlal Nehru Medical College, Datta Meghe Institute of Higher Education and Rersearch, Wardha, IND; 3 Obstetrics and Gynaecology, Jawaharlal Nehru Medical College, Datta Meghe Institute of Higher Education and Rersearch, Wardha, IND; 4 Nursing, Shalinitai Meghe College of Nursing, Datta Meghe Institute of Higher Education and Research, Wardha, IND; 5 Medicine and Surgery, Jawaharlal Nehru Medical College, Datta Meghe Institute of Higher Education and Research, Wardha, IND; 6 Research and Development, Jawaharlal Nehru Medical College, Datta Meghe Institute of Higher Education and Research, Wardha, IND

**Keywords:** prevention, regulation, public health, performance-enhancing substances, bodybuilding

## Abstract

The use of performance-enhancing substances in bodybuilding is a well-known and long-standing issue. This comprehensive review article provides a detailed overview of the history of performance-enhancing substance use in bodybuilding, the types of substances commonly used, and the short-term and long-term health effects associated with their use. Additionally, the article discusses the regulation of performance-enhancing substances in various countries and the role of healthcare professionals in preventing substance use. The article also highlights the impact of substance use on society and the importance of prevention and intervention strategies. Finally, the article emphasizes the role of policymakers in addressing performance-enhancing substance use, including the development of regulations, penalties for violating rules, and the provision of resources for prevention and intervention programs. Overall, this review article sheds light on the dark side of bodybuilding and provides insight into the public health implications of performance-enhancing substance use.

## Introduction and background

Bodybuilding is a sport that involves the rigorous training and development of the body's muscles through a combination of weightlifting, cardio, and nutrition. While it has been around for centuries, it gained popularity as a competitive sport in the 20th century, with the first Mr. Olympia contest in 1965. Since then, bodybuilding has become a global phenomenon with millions of enthusiasts and a billion-dollar industry [1,2]. One of the most controversial aspects of bodybuilding is using performance-enhancing substances (PES), including anabolic-androgenic steroids, human growth hormone, insulin, diuretics, stimulants, and others. Athletes use these substances to increase muscle mass, improve strength and endurance, and enhance their physical appearance. While PES use is not unique to bodybuilding, it is more prevalent in this sport than in most others [3,4].

Bodybuilding has a long history of performance-enhancing substance use. The early forms of PES in bodybuilding were simple remedies such as caffeine, alcohol, and opiates. However, the emergence of anabolic-androgenic steroids in the mid-20th century revolutionized the sport and transformed it into what we see today [5]. In the early days of bodybuilding, natural training methods and diet were the only ways to achieve a well-sculpted body. However, with the rise of the anabolic steroid industry in the 1950s, athletes started experimenting with testosterone and other substances to enhance their performance. By the 1960s, anabolic steroids were widely used in bodybuilding circles, rapidly increasing muscle mass and strength gains [6].

Since then, the use of performance-enhancing substances in bodybuilding has evolved dramatically. Athletes now use various substances, including human growth hormone, insulin, diuretics, stimulants, and others, to gain a competitive edge. Additionally, the methods for administering these substances have become more sophisticated, including intravenous injections, transdermal patches, and oral dosages [7]. The use of performance-enhancing substances in bodybuilding is driven by several factors, including the desire to achieve a competitive edge, the pursuit of the perfect physique, and the pressure to meet societal beauty standards. In addition, some athletes may feel that they need to use PES to keep up with others using them [8]. Moreover, using PES can also provide psychological benefits, such as increased confidence and self-esteem. For many athletes, bodybuilding is a way of life, and they are willing to go to great lengths to achieve their goals [9].

The objective of this review is to critically examine the use of performance-enhancing substances in the field of bodybuilding and shed light on the resulting implications for public health. By analyzing the prevalence, effects, motivations, and risks associated with using these substances, this article aims to understand the complex relationship between bodybuilding and performance-enhancing substances thoroughly. Through a comprehensive synthesis of current research and literature, the objective is to contribute to the existing knowledge base and promote informed discussions among professionals, policymakers, and the general public regarding the potential health consequences and broader societal implications of performance-enhancing substance use in the context of bodybuilding.

## Review

Types of performance-enhancing substances used in bodybuilding

Anabolic Steroids

Anabolic steroids are a class of synthetic substances that mimic the effects of the male hormone testosterone in the body. Bodybuilders and other athletes use these substances to increase muscle mass, strength, and endurance as well as to shorten recovery times after workouts. Anabolic steroids are also used medically to treat delayed puberty, muscle wasting, and osteoporosis [10]. Anabolic steroids are associated with numerous health risks, both short-term and long-term. Anabolic steroid use can cause acne, hair loss, and mood swings in the short term. It can also lead to liver damage, cardiovascular disease, and hormonal imbalances. Anabolic steroids can increase blood pressure and cholesterol levels, increasing the risk of heart attack and stroke. Additionally, they can cause the development of breast tissue in men, a condition known as gynecomastia [11].

Long-term use of anabolic steroids can lead to even more serious health consequences. It can cause kidney damage and increase the risk of prostate cancer. It can also lead to infertility, impotence, and reduced testicular size. Anabolic steroid use can also cause psychological effects, including mood swings, aggression, and depression [12]. In addition to the health risks associated with anabolic steroid use, legal and ethical concerns exist. Anabolic steroids are banned by most sports organizations, including the International Olympic Committee (IOC) and the World Anti-Doping Agency (WADA). These substances are illegal in many countries, including the United States [13].

Human Growth Hormone

Human growth hormone (HGH) is a hormone naturally produced by the pituitary gland and plays a crucial role in stimulating growth, cell reproduction, and regeneration in humans. It is important in childhood for normal growth and development, and in adulthood, it helps to maintain muscle mass and bone density and regulate metabolism. HGH also significantly affects the immune system, cognitive function, and mood [14]. HGH is a performance-enhancing substance used in bodybuilding to increase muscle mass, reduce body fat, and improve athletic performance. The use of HGH in bodybuilding is often combined with other substances, such as anabolic steroids, to achieve greater results [15]. However, using HGH in bodybuilding is associated with various health risks. One of the most significant risks is the development of diabetes, as HGH use can cause insulin resistance and impaired glucose tolerance. Other side effects of HGH use include joint pain, carpal tunnel syndrome, and an increased risk of heart disease and stroke [10,12,15].

Furthermore, studies have shown that long-term HGH use may increase cancer risk, particularly in the digestive system, such as colon, pancreas, and stomach cancer. This risk is likely due to the growth-promoting effects of HGH, which may stimulate the growth of cancerous cells [1,6,15]. It is important to note that using HGH in bodybuilding is illegal without a prescription and is classified as a controlled substance in many countries. The misuse of HGH can result in serious health consequences, and it is important to raise awareness about the risks associated with its use in bodybuilding. Healthcare professionals should also be vigilant about using HGH and other performance-enhancing substances in their patients and work towards preventing and treating the associated health issues [6,8].

Insulin

Insulin is a hormone that plays a crucial role in regulating blood sugar levels. The pancreas produces it, and its primary function is to help cells in the body absorb glucose from the bloodstream, which is then used for energy. In bodybuilding, insulin is often used as a performance-enhancing substance due to its anabolic effects. Insulin stimulates the uptake of amino acids and glucose into muscle cells, promoting muscle growth and repair [16]. However, the misuse of insulin can have serious health consequences. When insulin is used improperly or excessively, it can cause hypoglycemia, a dangerous condition characterized by low blood sugar levels. Hypoglycemia can cause dizziness, confusion, sweating, and even loss of consciousness. In severe cases, it can lead to seizures, coma, and even death [17,18].

Another potential danger of insulin misuse is the development of insulin resistance, a condition where cells become less responsive to the effects of insulin. This can lead to elevated blood sugar levels and the development of type 2 diabetes [19]. In addition to these health risks, using insulin in bodybuilding is also illegal and prohibited by most sports organizations. Its use is considered doping and can result in disqualification, suspension, and other penalties. Therefore, it is important for athletes to understand the risks associated with insulin misuse and to avoid using it as a performance-enhancing substance [20].

Diuretics

Diuretics are drugs that increase the rate of urine production by the kidneys, resulting in a loss of water and electrolytes from the body. In bodybuilding, diuretics are sometimes used to help reduce water retention in the body and promote a more defined and muscular appearance on stage. The use of diuretics in this manner is sometimes referred to as "cutting water weight" [21]. However, the misuse of diuretics can have serious health consequences. By increasing the rate of urine production, diuretics can cause dehydration and electrolyte imbalances, leading to muscle cramps, dizziness, fainting, and even death in severe cases. The loss of electrolytes such as sodium and potassium can also negatively affect the heart, leading to arrhythmias and other cardiovascular complications [22].

Moreover, the use of diuretics can also put a significant strain on the kidneys. The kidneys are responsible for filtering the blood and removing waste products, and diuretics can increase the workload on these organs. Long-term use of diuretics can lead to kidney damage, impairing their ability to function properly and ultimately resulting in kidney failure [23]. In addition to these health risks, using diuretics in bodybuilding is prohibited by many athletic organizations and can result in disqualification and other penalties. Therefore, bodybuilders need to avoid the misuse of diuretics and instead focus on healthy and sustainable approaches to achieving their desired physique [24].

Stimulants

Stimulants are a class of performance-enhancing substances some bodybuilders use to increase focus, alertness, and energy during their workouts. Some of the most commonly used stimulants in bodybuilding include amphetamines, caffeine, ephedrine, and clenbuterol. These substances are believed to enhance mental and physical performance, reduce fatigue, and improve endurance [25]. While stimulants can provide short-term benefits, their misuse can have serious health consequences. For instance, using stimulants can cause an increase in heart rate and blood pressure, leading to cardiovascular problems such as heart attack, stroke, and arrhythmia. Additionally, the misuse of stimulants can lead to addiction and withdrawal symptoms, including anxiety, depression, and insomnia [26].

Caffeine, found in many beverages and dietary supplements, is the most widely used stimulant in bodybuilding. While caffeine is generally safe in moderation, excessive use can lead to negative side effects, including restlessness, nervousness, and insomnia. Caffeine can also increase heart rate and blood pressure and cause gastrointestinal problems such as nausea and diarrhea [27]. Ephedrine and clenbuterol are also commonly used stimulants in bodybuilding. Ephedrine is a central nervous system stimulant known to increase metabolic rate and fat burning. Still, its use has been associated with serious health risks such as high blood pressure, heart attack, and stroke. Similarly, clenbuterol, often used to promote weight loss, has been linked to heart problems and muscle tremors [28].

Short-term and long-term health effects of performance enhancing substance use in bodybuilding

Short-Term Effects

Acne: Anabolic steroids can cause acne, particularly on the face, chest, and back. This is because steroids increase sebum production, which can clog the pores and lead to the development of acne. In severe cases, acne can become infected and lead to scarring [29].

Baldness: Anabolic steroids can cause hair loss, particularly in men genetically predisposed to baldness. This is because steroids can increase the production of dihydrotestosterone (DHT), a hormone that causes hair follicles to shrink and eventually stop producing hair [30].

Liver damage: Anabolic steroids can cause liver damage, including liver tumors and cysts. Recognizing that the reported liver damage, including liver tumors and cysts, is associated with such misuse is crucial. Steroids are metabolized in the liver, and prolonged or inappropriate usage can lead to liver complications. In severe cases, liver damage may necessitate a liver transplant [31].

Cardiovascular problems: Performance-enhancing substances can increase blood pressure and cholesterol levels, leading to cardiovascular problems such as heart attacks and strokes. Anabolic steroids can also cause the heart to enlarge, which can increase the risk of heart failure and lethal arrhythmias. Additionally, using stimulants such as ephedrine can cause irregular heartbeats and increase the risk of heart attacks [32]. It is important to note that the severity of these short-term effects can vary depending on the type and number of performance-enhancing substances used and individual factors such as age, sex, and overall health. However, these short-term effects can be serious and potentially life-threatening and should not be taken lightly.

Long-Term Effects

Kidney damage: The kidneys are responsible for filtering waste and excess fluids from the body. Prolonged use of performance-enhancing substances such as anabolic steroids, diuretics, and growth hormones can lead to kidney damage and failure. The excessive workload on the kidneys can cause them to become enlarged, leading to kidney stones and other complications [33].

Infertility: Performance-enhancing substances, particularly anabolic steroids, can disrupt the normal production of hormones in the body, leading to infertility in both men and women. Anabolic steroid use can reduce testosterone production in men, decreasing sperm count and motility. In women, anabolic steroids can disrupt the menstrual cycle and cause infertility [34].

Cardiovascular problems: Prolonged use of performance-enhancing substances can increase the risk of cardiovascular problems, including heart disease and stroke. Anabolic steroids can cause an increase in LDL cholesterol (the "bad" cholesterol) and a decrease in HDL cholesterol (the "good" cholesterol), which can lead to the buildup of plaque in the arteries. This can increase the risk of heart attack and stroke [35].

Increased risk of cancer: Some performance-enhancing substances have been linked to increased cancer risk. Anabolic steroids have been linked to an increased risk of liver, kidney, and prostate neoplasms. Growth hormone has been linked to an increased risk of colon cancer [36]. It is important to note that the health effects of performance-enhancing substances can vary depending on the substance used, the dosage, and the duration of use. In some cases, the health effects may be reversible if the substance is discontinued. However, in many cases, the damage caused by these substances can be permanent and life-threatening.

The regulation of performance-enhancing substances in bodybuilding

The Role of Regulatory agencies

Regulatory agencies, such as the WADA and the IOC, regulate performance-enhancing substances in sports, including bodybuilding. These agencies play a critical role in ensuring fair competition and protecting the health of athletes [37]. WADA was established in 1999 and is responsible for promoting, coordinating, and monitoring the fight against doping in sports. WADA has developed a list of prohibited substances and methods in sports, including anabolic steroids, growth hormones, insulin, and diuretics. The list is updated annually and is recognized by all major international sports organizations, including the IOC [37].

The IOC, on the other hand, is responsible for organizing and overseeing the Olympic Games. The IOC also works closely with WADA to ensure that athletes who compete in the Olympics comply with anti-doping regulations. The IOC has a zero-tolerance policy towards doping and works with national anti-doping organizations to enforce anti-doping regulations [37]. In addition to WADA and the IOC, there are also national anti-doping agencies (NADAs) responsible for enforcing anti-doping regulations at the national level. These agencies work with WADA and the IOC to implement anti-doping programs and policies [38]. The role of regulatory agencies in bodybuilding is essential, as it ensures that athletes compete on a level playing field and that their health and safety are protected. However, the effectiveness of current regulations and enforcement efforts has been questioned, and there are concerns about the widespread use of performance-enhancing substances in bodybuilding despite anti-doping regulations. More must be done to strengthen regulation and enforcement efforts and educate athletes about the risks and consequences of using these substances [39].

The Current State of Regulations

The current state of regulations on using performance-enhancing substances in bodybuilding is complex and varied across different countries and regions. While there are regulatory agencies that aim to control the use of these substances, such as the WADA and the U.S. Anti-Doping Agency (USADA), their efforts have not been entirely successful in eliminating the use of these substances in bodybuilding [40]. One of the challenges in regulating the use of performance-enhancing substances is the availability of these substances through black market sources. Many bodybuilders and fitness enthusiasts can obtain these substances outside of legal channels, making it difficult for regulatory agencies to track and control their distribution [41].

Additionally, there is a lack of oversight and regulation in the manufacturing and distribution of these substances, which can lead to the production of unsafe and contaminated products. This can increase the health risks associated with their use and further complicate efforts to regulate them [42]. Overall, the current state of regulations related to performance-enhancing substance use in bodybuilding is insufficient. There is a need for more coordinated and effective efforts to address this issue, including increased oversight of manufacturing and distribution, greater awareness and education among bodybuilders and fitness enthusiasts, and stronger penalties for those who violate regulations [43].

The Effectiveness of Current Regulations

The effectiveness of current regulations in curbing the use of performance-enhancing substances in bodybuilding is a topic of debate among experts in the field. While some argue that the penalties for using these substances are not severe enough to deter athletes from using them, others argue that the testing methods used by regulatory agencies are not always accurate or effective in detecting the use of these substances [44]. In many countries, performance-enhancing substances are classified as controlled substances, and their use and distribution are strictly regulated. However, some athletes still obtain these substances through the black market as undetectable or masking agents. Furthermore, there is a lack of consensus among regulatory agencies regarding the severity of penalties for using these substances, with some imposing more stringent penalties than others [45,46].

Another issue with the effectiveness of current regulations is the lack of education and awareness among bodybuilders and fitness enthusiasts about the risks associated with using these substances. Many athletes are unaware of the potential health consequences of using performance-enhancing substances, and some may even believe these substances are safe to use. More must be done to educate athletes and the general public about the dangers of performance-enhancing substance use in bodybuilding and encourage safe and healthy sports practices [47,48].

Public health implications

Legal Status of Performance-Enhancing Substances in Various Countries

The use and possession of performance-enhancing substances such as anabolic steroids, growth hormones, insulin, and diuretics are regulated differently worldwide. In some countries, these substances are legal only with a prescription, while in others, they are entirely illegal and can result in severe legal consequences [49]. For example, in the United States, anabolic steroids are classified as Schedule III controlled substances. Their use and possession without a valid prescription are considered federal crimes, leading to significant fines and prison time. Similarly, in the UK, using anabolic steroids without a prescription is illegal and can result in prosecution, with a maximum sentence of 14 years in prison [50].

However, it's worth noting that certain countries have more lenient laws regarding the use of performance-enhancing substances. In Mexico and Thailand, anabolic steroids are legal and can be purchased over the counter without a prescription. In Brazil, anabolic steroids are legal, but their purchase requires a prescription from a licensed physician [51]. The legal status of other performance-enhancing substances, such as growth hormones and insulin, also varies from country to country. In the United States, growth hormone is legal only with a prescription for specific medical conditions. In contrast, it is legal for anti-aging purposes in some European countries, like Germany and France. Similarly, insulin is legal only with a prescription in most countries, but in others, like the UK and Canada, it can be purchased over the counter without a prescription [52].

Role of Healthcare Professionals in Preventing Performance-Enhancing Substance Use in Bodybuilding

Healthcare professionals are critical to preventing performance-enhancing substances in bodybuilding and safeguarding public health. They can educate their patients about the risks associated with these substances, including short-term and long-term health effects. Additionally, healthcare professionals can provide information on safe and effective training and nutrition strategies to help individuals achieve their fitness goals healthily and sustainably [53]. Assessing the patient's health status is one of the key responsibilities of healthcare professionals. They can offer guidance on the importance of a balanced and nutritious diet, regular exercise, and adequate rest. Moreover, healthcare professionals can provide information on safe and effective training techniques and help patients set realistic and achievable goals [54].

Healthcare professionals are also well-positioned to identify individuals at risk for performance-enhancing substance use. This includes individuals with body image issues, low self-esteem, or a history of substance abuse. By addressing underlying psychological or emotional issues contributing to the desire to use these substances, healthcare professionals can provide crucial support and guidance [55]. Furthermore, healthcare professionals can collaborate with regulatory agencies and policymakers to develop and implement effective prevention and intervention strategies. They can advocate for stricter regulations, increase public awareness campaigns, and work with sports organizations to promote safe and healthy practices in bodybuilding [56].

Impact of Performance-Enhancing Substance Use on Society

The impact of performance-enhancing substance use extends beyond individual health consequences and significantly affects the broader community. One major concern is that the use of these substances promotes a culture of cheating and unfair competition, eroding trust in sports and other competitive arenas. When individuals use performance-enhancing substances to gain an unfair advantage, it undermines the integrity of the competition. It diminishes the achievements of athletes who have worked hard without using such substances [57]. Moreover, using performance-enhancing substances sends a dangerous message to young people that these substances are necessary for success in sports or life. This message can lead to unhealthy behavior patterns and attitudes towards sports and physical activity. Young individuals may feel pressured to use these substances to keep up with their peers or excel in their chosen sport, which can result in serious health consequences [58,59].

Furthermore, the pressure to use performance-enhancing substances can extend beyond individual athletes to the broader athletic community. Other athletes may feel compelled to use these substances to remain competitive, creating a dangerous substance use and abuse cycle. This culture of dependency on performance-enhancing substances can have long-lasting and far-reaching effects on the health and well-being of individuals and communities [60].

Prevention and intervention strategies

Available Prevention and Intervention Strategies

Prevention and intervention strategies can effectively prevent and address the use of performance-enhancing substances in bodybuilding. One important strategy is education and awareness programs focusing on the risks associated with performance-enhancing substance use and safe and effective training and nutrition strategies. Education and awareness programs can be directed toward athletes, coaches, trainers, parents, and the general public. These programs can be delivered through various channels, including social media, websites, schools, sports clubs, and community organizations [61]. Another strategy is to develop and implement regulations that limit access to these substances and penalize those who violate the rules. Regulatory measures can include drug testing, bans on certain substances, and penalties for those who violate the rules. This can be done at various levels, including international, national, and local organizations. The regulations can also involve the cooperation of different stakeholders, such as sports organizations, government agencies, and law enforcement [62].

Interventions may include counseling, behavioral therapies, and pharmacological treatments to help individuals overcome substance abuse and addiction. Counseling and behavioral therapies can help individuals address underlying issues contributing to substance abuse, such as stress, anxiety, or depression. These interventions can help individuals develop coping skills, improve communication, and strengthen social support networks. Pharmacological treatments may also help individuals manage withdrawal symptoms and cravings associated with substance abuse [63]. Furthermore, policymakers can play a significant role in addressing the dark side of bodybuilding by funding research on performance-enhancing substance use and its effects and developing policies promoting safe and healthy sports practices. Policies can include the development of guidelines for athletes, coaches, and trainers, as well as implementing prevention and intervention programs in schools and sports clubs [64].

Importance of Education and Awareness

Education and awareness programs are essential to preventing the use of performance-enhancing substances in bodybuilding. These programs can give athletes the necessary knowledge to make informed decisions about their health and performance goals. By educating athletes on the potential risks and negative health consequences associated with performance-enhancing substances, they can make informed decisions that prioritize their health and well-being [65]. Moreover, education and awareness programs can help dispel myths and misconceptions about performance-enhancing substances, leading to safer and healthier practices among athletes. For example, athletes may believe using performance-enhancing substances is the only way to achieve their desired physique or performance goals. However, with the right education and awareness, athletes can learn about alternative, safe, and effective strategies for achieving their goals without compromising their health [66].

Education and awareness programs can also promote a culture of fair competition and sportsmanship. When athletes know the risks and consequences associated with performance-enhancing substance use, they are more likely to avoid using these substances and compete on a level playing field. This can lead to a healthier and more positive sporting culture that values safe and fair competition over winning at all costs [67].

Role of Policymakers in Addressing Performance-Enhancing Substance Use in Bodybuilding

The role of policymakers in addressing performance-enhancing substance use in bodybuilding cannot be overstated. Policymakers can help ensure that performance-enhancing substances are not widely available and that access to them is limited. This can be done by developing and implementing regulations restricting these substances' sale and distribution. For example, some countries have laws requiring individuals to have a prescription before purchasing performance-enhancing substances such as anabolic steroids. Other countries have banned the sale and distribution of these substances entirely [57]. In addition to regulations, policymakers can also develop penalties for individuals who violate the rules. These penalties may include fines, suspension from competition, or even legal charges. By making it clear that the use of performance-enhancing substances is unacceptable, policymakers can help deter individuals from using these substances in the first place [68].

Policymakers can also provide resources for prevention and intervention programs. These programs can educate athletes about the risks associated with performance-enhancing substances and provide information about safe and healthy ways to achieve their goals. Prevention and intervention programs can also provide support and resources for individuals struggling with substance use issues [69]. Finally, policymakers can support research to better understand the risks associated with performance-enhancing substances and develop new, more effective prevention and intervention strategies. By supporting research, policymakers can help ensure that athletes have access to the best possible information and resources to help them make informed decisions about their health and well-being [70].

## Conclusions

In conclusion, this review article has discussed the dark side of bodybuilding, specifically the use of performance-enhancing substances and their public health implications. The article has highlighted the history and evolution of performance-enhancing substance use in bodybuilding, the types of substances used, and their short-term and long-term health effects. The article has also discussed the regulation of performance-enhancing substances, the legal status of these substances in different countries, and the role of healthcare professionals and policymakers in preventing their use. Prevention and intervention strategies have also been discussed, including the importance of education and awareness, regulation, and intervention strategies. It is important to address the dark side of bodybuilding and the use of performance-enhancing substances because they pose a significant threat to public health. The use of these substances can lead to serious short- and long-term health consequences and contribute to the development of an unhealthy and dangerous culture of competition. The current state of regulations and prevention efforts is insufficient, and more must be done to address this issue. Future research in this area should focus on developing more effective prevention and intervention strategies and a better understanding of the short-term and long-term health effects of performance-enhancing substance use. Research should also explore the social and cultural factors that contribute to the use of these substances and how they can be addressed. Overall, a comprehensive and coordinated effort is needed to address the dark side of bodybuilding and promote safe and healthy practices in this sport.
